# Profiling of the full-length transcriptome in abdominal aortic aneurysm using nanopore-based direct RNA sequencing

**DOI:** 10.1098/rsob.210172

**Published:** 2022-02-02

**Authors:** Hai Xin, Xingqiang He, Jun Li, Xiaomei Guan, Xukui Liu, Yuewei Wang, Liyuan Niu, Deqiang Qiu, Xuejun Wu, Haofu Wang

**Affiliations:** ^1^ Department of Vascular Surgery, Shandong Provincial Hospital, Cheeloo College of Medicine, Shandong University, Jinan, Shandong 250021, People's Republic of China; ^2^ Department of Cardiology, Xijing Hospital, Fourth Military Medical University, 169 West Changle Road, Xi'an 710032, People's Republic of China; ^3^ Department of Vascular Surgery, The Affiliated Hospital of Qingdao University, Qingdao, Shandong 266000, People's Republic of China

**Keywords:** abdominal aortic aneurysm, nanopore-based RNA sequencing, alternative splicing, alternative polyadenylation, lncRNAs

## Abstract

Abdominal aortic aneurysm (AAA) is a common and serious disease with a high mortality rate, but its genetic determinants have not been fully identified. In this feasibility study, we aimed to elucidate the transcriptome profile of AAA and further reveal its molecular mechanisms through the Oxford Nanopore Technologies (ONT) MinION platform. Overall, 9574 novel transcripts and 781 genes were identified by comparing and analysing the redundant-removed transcripts of all samples with known reference genome annotations. We characterized the alternative splicing, alternative polyadenylation events and simple sequence repeat (SSR) loci information based on full-length transcriptome data, which would help us further understand the genome annotation and gene structure of AAA. Moreover, we proved that ONT methods were suitable for the identification of lncRNAs via identifying the comprehensive expression profile of lncRNAs in AAA. The results of differentially expressed transcript (DET) analysis showed that a total of 7044 transcripts were differentially expressed, of which 4278 were upregulated and 2766 were downregulated among two groups. In the KEGG analysis, 4071 annotated DETs were involved in human diseases, organismal systems and environmental information processing. These pilot findings might provide novel insights into the pathogenesis of AAA and provide new ideas for the optimization of personalized treatment of AAA, which is worthy of further study in subsequent studies.

## Graphical abstract

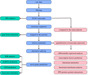

## 1. Introduction

Abdominal aortic aneurysm (AAA), as a common and severe disease associated with high mortality, is characterized by a progressive segmental abdominal aortic dilation [1]. Usually, the non-ruptured aneurysms are asymptomatic unless clinical complications occur, which often induces acute rupture or thromboembolism and result in a lethal rate of up to 85%; therefore, its clinical assessment is challenging [2,3]. However, there is still no effective pharmacological treatment to control its progression or the risk of rupture. Clinically, surgical intervention, including open or endovascular aortic repair of the dilated aorta, remains the only reliable treatment option [4–6]. Therefore, it is important to better understand the mediators and mechanism networks of AAA pathogenesis to identify novel therapeutic targets.

Over the past decade, a large number of discoveries in human genetics related to complex diseases have been made through genome-wide association studies (GWAS) [7]. In addition, emerging evidence has shown that genetic variants are strongly associated with a number of cardiovascular diseases through GWAS studies, including AAA, coronary artery disease, myocardial infarction, as well as vascular remodelling, blood pressure, triglyceride cholesterol and LDL metabolism [8,9]. Previous studies have shown that genetic components account for approximately 70% of total AAA susceptibility [10], suggesting genetic factors play a vital role in aetiology. However, the genetic determinants of AAA have not yet been fully determined.

**Figure 1.**
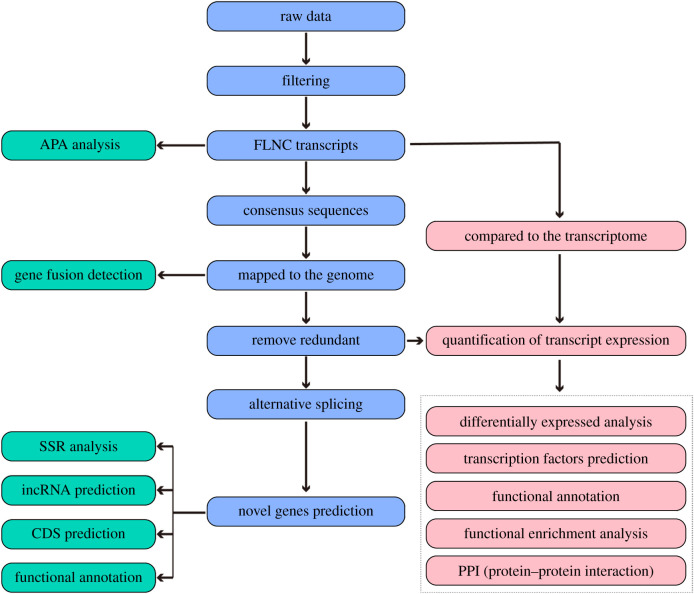
Study design illustration.

In the present paper, the transcriptome characterization of AAA was identified by the Oxford Nanopore Technologies (ONT) MinION platform, and its possible molecular mechanism was further revealed. By analysing transcriptome data, we attempted to reveal the vital transcripts and pathways implicated in AAA. The study design is presented in figure 1. These results will help to provide critical insights into the pathogenesis of AAA for future searches of the therapeutic targets.

## 2. Material and methods

### 2.1. Specimen gathering

AAA samples were obtained from five patients undergoing open surgical treatment, and another five peripheral blood samples of healthy subjects were gathered as the control group (t group) in the affiliated hospital of Qingdao University from between January 2019 and January 2020.

### 2.2. RNA extraction, cDNA library preparation and nanopore sequencing

Total RNA was isolated from the samples using Trizol reagents (Invitrogen, Carlsbad, CA, USA). One microgram of total RNA was prepared for the cDNA library construction by the cDNA-PCR sequencing kit (SQK-PCS109, Oxford Nanopore Technologies, Oxford, UK). Finally, the cDNA libraries within the FLO-MIN109 flow cells were worked on the PromethION platform (Biomarker Technology Company, Beijing, China). These testing processes were conducted according to the protocol of the manufacturer.

### 2.3. Remove redundant and find fusion transcript

Minimap2 program was employed to match the consensus sequences to the reference genome. Then, using the cDNA Cupcake package with min-coverage = 85% and min-identity = 90%, the mapped reads were collapsed. When collapsing redundant transcripts, the 5′ difference was not taken into account.

The criteria for fusion candidate genes were the single transcript, with (i) minimum distance of 10 kb between the loci, (ii) total coverage of ≥ 95%, (iii) minimum coverage rate of per loci was 5% and minimum coverage (bp) ≥ 1 bp, and (iv) at least map to 2 or more loci.

### 2.4. Structural analysis of transcripts

Gffcompare was used to verify transcripts to the annotated transcripts of known reference. AStalavista and TransDecoder tools were employed to identify the alternative splicing (AS), such as intron retention (IR), exon-skipping (ES), AD, AA and MEE, and alternative polyadenylation (APA) events, respectively. CDSs and simple sequence repeat (SSR) analysis of transcriptome was performed by TransDecoder and MISA program, respectively.

### 2.5. LncRNA analysis

In the transcripts, the criteria of the minimum length greater than or equal to 200 nucleotides and at least 2 exon count thresholds were applied to screen the lncRNA candidates. Then, lncRNAs were further distinguished by four computational methods combined, including coding potential assessment tool (CPAT), protein family (Pfam), coding-non-coding index (CNCI) and coding potential calculator (CPC).

### 2.6. Annotation of functionality and enrichment analysis

Gene function was annotated based on the following databases: NR (NCBI non-redundant protein sequences, ftp://ftp.ncbi.nih.gov/blast/db/), Pfam (Protein family, http://pfam.xfam.org/), KOG (http://www.ncbi.nlm.nih.gov/KOG/), COG (http://www.ncbi.nlm.nih.gov/COG/), eggNOG (http://eggnogdb.embl.de/), KEGG (Kyoto Encyclopedia of Genes and Genomes, http://www.genome.jp/kegg/) and GO (Gene Ontology, http://www.geneontology.org/). The KOBAS software was used to test the statistical enrichment of differential expression genes in KEGG pathways.

### 2.7. Quantification and differential expression analysis of gene/transcript expression

The full-length sequencing transcriptome and publicized genomic transcripts of reference were used for sequence alignment. The match quality of reads greater than 5 was further quantified. The expression level was evaluated via reads per gene/transcript mapped per 10 000 reads.

The DESeq R package (1.18.0) was used to conduct differential expression analysis of two conditions/groups for the specimens with biological replicates. For controlling the false discovery rate, Benjamini and Hochberg's approach was employed to adjust the resulting *p*-values. Genes screened by DESeq with FDR < 0.05 and fold change ≥ 2 were specified as differentially expressed.

To specimens with no biological duplicates, read counts for each sequenced library were adjusted via edgeR program package prior to differential gene expression analysis. The EBSeq R package (1.6.0) was used for differential expression analysis of two samples and the PPDE (posterior probability of being DE) for the resulting FDR (false discovery rate) adjustment. Threshold with FDR < 0.05 and foldchange ≥ 2 was considered to be significantly differential expression.

### 2.8. Protein–protein interaction

Based on the results of differential expression analysis, the predicted PPI of these differentially expressed transcripts (DETs) were obtained by the blast the sequences of the DETs with the genome of a related species (the protein interaction of which exists in the STRING database: http://string-db.org/). Then, the Cytoscape program was used to visualize the PPI of these DEGs.

## 3. Results

### 3.1. ONT sequencing overview

Overall, we constructed 10 transcriptome libraries in total (b1–b5 of the AAA group and t1–t5 of the control group), and 2.71 GB of clean data was output in each sample. After discarding the low-quality and short reads, 19 680 639 and 16 012 762 clean reads were obtained from the AAA and control group, with a mean N50 of 768 and 1241.8 bp, and the average read length of 820.8 and 1125 bp, respectively (electronic supplementary material, table S1 and figure S1). Additionally, the quality (Q) score distribution of the above 10 groups of raw reads was distributed between Q6 and Q18, with Q12 and Q13 accounting for the highest percentage (electronic supplementary material, figure S2). 13 977 197 and 10 088 571 clean reads were generated after removing rRNA, among which 88.07% and 84.18% were identified to be full length, respectively (electronic supplementary material table S2 and figure S3). Then, 1 to 24 fusion transcripts were obtained from each sample (electronic supplementary material, file S1). In total, 9574 novel transcripts and 781 genes were identified through comparing and analysing the redundant-removed transcripts of all samples with known reference genome annotation (electronic supplementary material, files S2 and S3).

### 3.2. Characterization of alternative splicing, alternative polyadenylation and SSR

**Figure 2.**
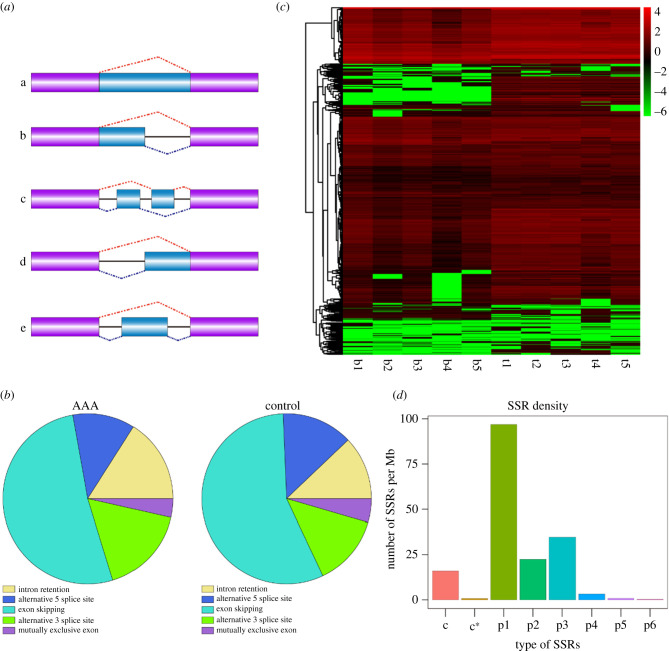
Identifying and characterizing AS, APA events and SSR loci information. (*a*) AS of different types. a: intron retention; b: alternative 5′ splice site; c: alternative exon; d: alternative 3′ splice site; e: exon-skipping. (*b*) AS event distribution in two groups. (*c*) Heatmap of AS events corresponding to transcripts. (*d*) Distribution of different SSR types of specific SSR identifying in ONT.

Within the ONT data, a total of 13 427 AS events were detected and grouped into five classes, including 7339 ES events, 1976 alternative 3′ sites (Alt. 3′), 1827 IR events, 1730 alternative 5′ sites (Alt. 5′) and 555 mutually exclusive exon events (figure 2*a,b*). DETs with different AS events were of further concern (figure 2*c*). The identified APA of each sample is shown in the electronic supplementary material, figure S4, and the motif analysis of 50 bp sequences upstream of the polyA site of all transcripts is shown in electronic supplementary material, figure S5. In addition, transcripts longer than 500 bp were selected for SSR analysis by MISA. The result showed that a total of 38 844 SSRs were detected from ONT data (electronic supplementary material, file S4). SSR loci repeat units were 1 to 6 bases, of which the most frequent SSRs identified were p1 (21 458), followed by p3 (7700), p2 (4981), p4 (731) and p5 (182); few P6 (69) were discovered. Additionally, compound SSR and compound SSR with overlapping positions were 3558 and 165, respectively (figure 2*d*).

### 3.3. Coding sequence of novel genes, transcription factor and lncRNAs prediction

**Figure 3.**
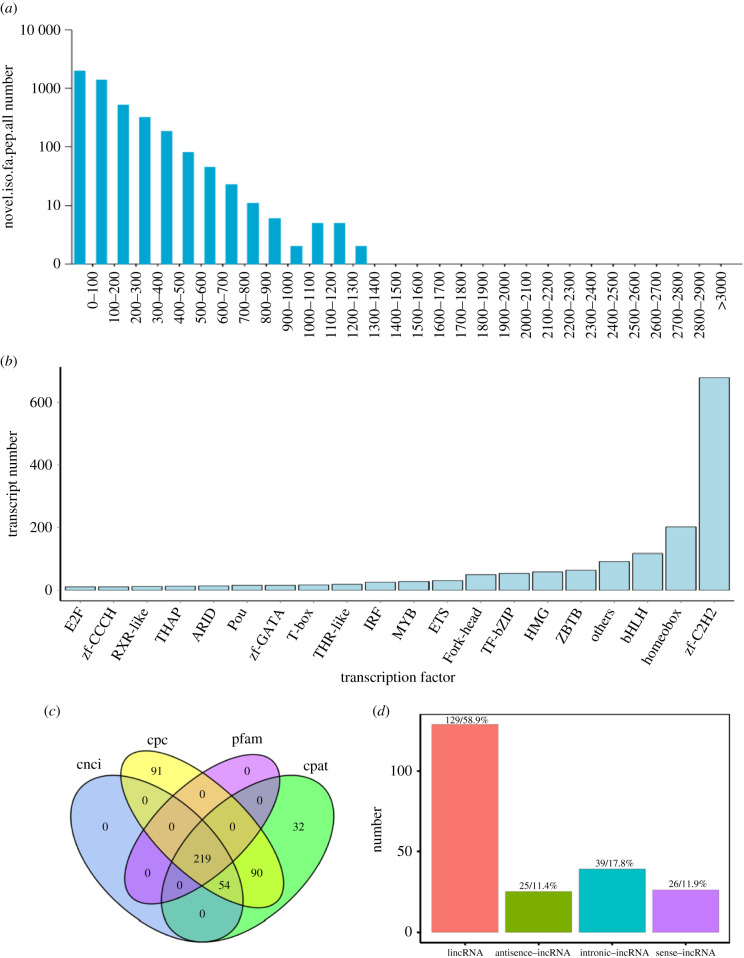
The distribution map of predicted CDS-coding protein length, transcription factor types and characterization of long non-coding RNAs (lncRNAs) were determined. (*a*) Length distributions of the CDS lengths of complete ORFs. (*b*) Distribution of transcription factor types. (*c*) Venn diagrams displaying the counts of candidate lncRNAs filtered by CNCI, CPC, CPAT and Pfam result. (*d*) Map of lncRNA position classification.

Seven thousand and seven hundred ORFs were detected in the ONT data by TransDecoder (v.3.0.0), of which 4509 were complete ORFs. The CDS length distribution of the complete ORFs is shown in figure 3*a*, mostly ranged from 100 to 1400 aa. Additionally, the transcription factors were predicted for the new transcripts. The result revealed that 1772 transcription factors were obtained in total, and the different types of transcription factors are shown in figure 3*b*. Using CPC, CNCI and CPAT analysis, there were 219 lncRNAs at the intersections which were visualized by the Venn diagram (figure 3*c*), and the classification of lncRNAs are mapped in figure 3*d*.

### 3.4. Dynamic expression of transcripts in abdominal aortic aneurysm

**Figure 4.**
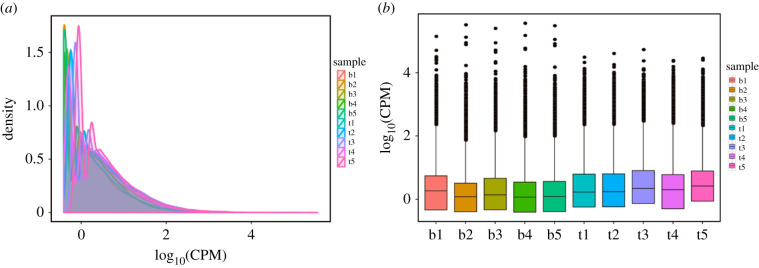
The overall distribution of transcript expressions of samples. (*a*) Comparison map of CPM density distribution of each sample. (*b*) Boxplot of CPM of each sample.

To get the annotation information of the transcripts, the obtained novel transcript sequences were aligned to the databases, such as NR, COG, KOG, Swissprot, GO, Pfam and KEGG. Functional annotation was conducted on the novel transcripts (electronic supplementary material, file S5), and the quantity of transcripts annotated in each database is listed in electronic supplementary material, table S3. The full-length sequencing transcriptome and known transcriptome of genome were used as a reference for sequence alignment and subsequent analysis. A matching profile between the transcriptome and the reference transcriptome was obtained using minimap2 to align full-length reads with the reference transcriptome. The count reads were aligned to the reference transcriptome, and the alignment statistics are shown in electronic supplementary material, table S4. The overall distribution of the expression level of sample transcripts is shown in figure 4*a*. To further examine the dispersion degree of expression level distribution of transcripts in a sample, and visually compare the overall transcripts expression level of different samples, counts per million (CPM) distribution was displayed by boxplot (figure 4*b*).

**Figure 5.**
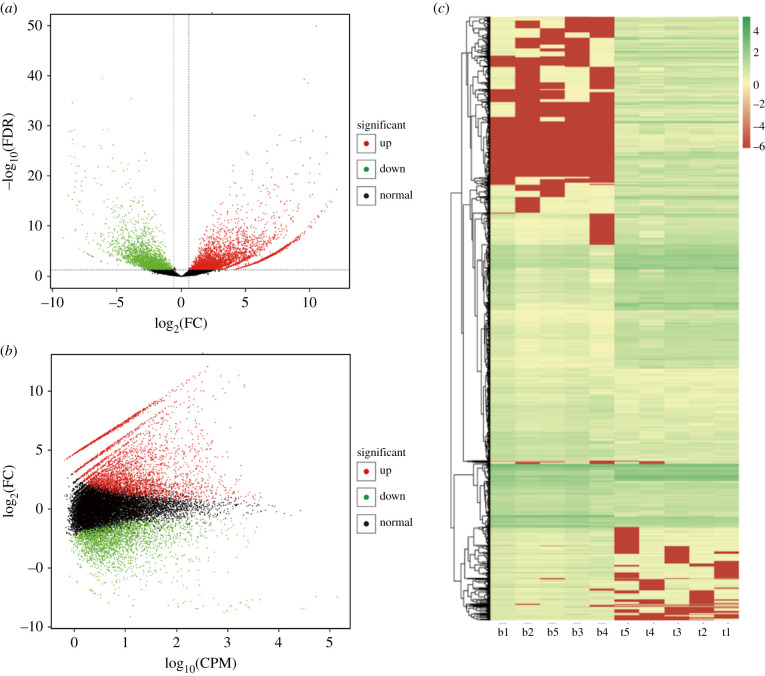
Dynamic expression of transcripts in AAA. (*a*) Volcano plot of DETs. (*b*) MA plot of DETs. (*c*) Clustering map of DETs.

In general, the expression of transcripts is temporal and spatially specific. DETs were defined as those with significantly different expression levels under two different conditions. DET analysis was carried out and results showed 7044 transcripts in total were differentially expressed, of which 4278 were upregulated and 2766 were downregulated. The volcano plot of differential expression is shown in figure 5*a*. The overall distribution of expression level and fold change of transcripts of the two groups of transcripts can be clearly seen through MA plot (figure 5*b*). Additionally, hierarchical clustering analysis was used to screen DETs and significant differences were found in their expression profiles (figure 5*c*).

### 3.5. Functional annotation of differentially expressed transcripts in abdominal aortic aneurysm

**Figure 6.**
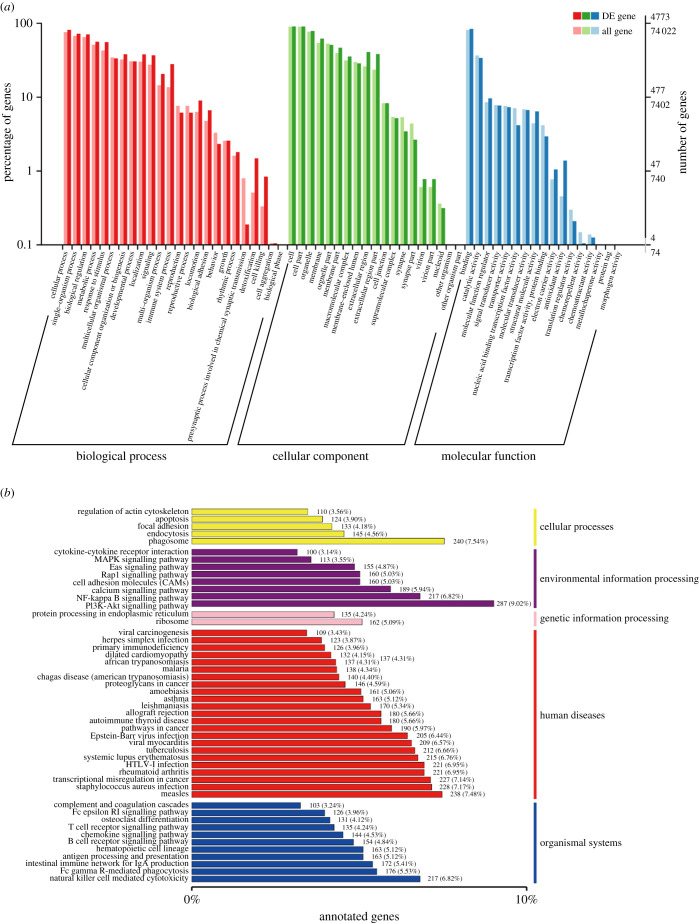
GO and KEGG annotation and enrichment of DETs. (*a*) GO annotation and classification of DETs. (*b*) KEGG classification figure of DETs.

**Figure 7.**
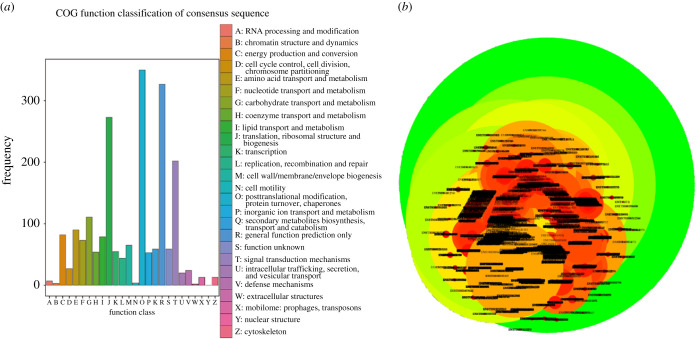
COG classification and PPI network of DETs. (*a*) The statistical figure of COG annotation and classification of DETs. (*b*) PPI network of DET protein.

The database function annotation for DETs was further executed, and the statistics of annotated transcripts number is listed in electronic supplementary material, table S5. The GO enrichment analysis on DETs was introduced (figure 6*a*), which revealed a number of highly enriched biological processes, such as the single-organism process, cellular process, metabolic process and biological regulation. Enrichment for the cellular components of cell, cell part, organelle and membrane were also observed. The target genes were mainly involved in the regulation of binding, catalytic activity, molecular function regulator and signal transducer activity in molecular function. In the KEGG analysis, organismal systems, human diseases and environmental information processing were the top three categories that account for the highest proportions (figure 6*b*). Generally, a total of 4071 annotated DETs were associated with some pathways associated with human disease, in which infectious disease was significantly identified, such as *Staphylococcus aureus* infection, tuberculosis, Epstein–Barr virus infection and Herpes simplex infection. A total of 1684 DETs might participate in organismal systems-related pathways, in which the Fc gamma R-mediated phagocytosis, natural killer cell-mediated cytotoxicity and intestinal immune network for IgA production were the top three pathways. A total of 1381 DETs were classified as belonging to environmental information processing, in which the PI3 K-Akt, NF-kappa B and calcium signalling pathways were the top three. Then, to further clarify the molecular function of DETs from AAA, they were allocated to COG classification and separated into 26 specified categories (figure 7*a*). The results revealed that the top hits include ‘protein turnover, posttranslational modification and chaperones', ‘signal transduction mechanisms', and ‘ribosomal structure, translation and biogenesis’ in both groups. Additionally, the Cytoscape visualization of the DETs protein interaction network is shown in figure 7*b*.

## 4. Discussion

AAA is one of the most common causes of death and disability in cardiovascular disease, particularly in the elderly population, which imposes an exorbitantly high financial burden on society. Except for a small percentage of incidental findings through an ultrasound-based screening programme, clinical diagnosis is usually at an advanced stage [11,12]. The risk of developing AAA is now considered to be a combination of personal lifestyle, environmental factors, genetic factors, and some physiological parameters or disease conditions, such as tobacco smoking history, increased age, male sex, cholesterol level, obesity, trauma, acute or chronic infection, connective tissue or inflammatory diseases, and family history [2,13–16]. Importantly, genetic components account for approximately 70% of the total susceptibility to AAA according to some estimates, suggesting that genetic factors may play a key part in aetiology. Interestingly, several studies have also reported that the strong linkage of aneurysm rupture to family history of AAA [17,18]. Therefore, identifying the genetic foundations of AAA will provide insights into the pathogenesis of the disease, and ultimately guide early surveillance, diagnosis, intervention and clinical decision-making.

Recently, a great quantity of evidence has shown an association between AAA and several microorganism infections, and there is a growing interest in this line of aetiologic investigation [19,20]. In this study, we identified 9574 novel transcripts and 781 genes by third-generation nanopore-based RNA sequencing combined with emerging genomic technologies from 10 libraries, detected the dynamic expression of transcripts, and further performed function annotation and GO enrichment analysis for DETs. In addition, using KEGG analysis, infection-related pathways related to human diseases (such as *Staphylococcus aureus*, tuberculosis and Epstein–Barr virus infection) were found to be highly expressed in annotated DETs of AAA samples. Matsui & Hatta [21] have reported a case of AAA in a patient with dialysis-related methicillin-resistant *Staphylococcus aureus* bacteraemia. Pathologically, *Staphylococcus aureus* may attach to the damaged intima by producing dextran, thereby invading the highly calcified arterial wall and causing mycotic aneurysm during bacteraemia. Previous studies have shown that tubercle bacillus could infect aortic wall causing AAA. Although this is a particularly rare complication of tuberculosis, it usually ruptures easily and causes serious clinical events [22,23]. The infection or reactivation of Epstein–Barr virus could lead to a variety of lymphoproliferative diseases and other less frequent clinical complications, including haematologic malignancies, haematologic malignancies, coronary artery aneurysm, etc. [24]. Several case-report studies suggest that Epstein–Barr virus infection may be related to coronary artery aneurysm and abdominal aortic lesions [25–27]. Unfortunately, the pathophysiologic mechanisms are still unclear and need further exploration.

AS and APA of RNAs are two conventional approaches for producing isoform diversity, leading to the production of different proteins necessary to maintain biological traits and function [28,29]. It is reported that more than 90% of human multi-exon genes undergo AS and almost 20% of genes have APA sites loci in introns [30,31]. In a previous aortic aneurysm study, mRNA expression and AS analysis of the identified proteins revealed different fingerprints between the bicuspid and tricuspid groups in dilated and non-dilated aortic tissue, implying AS may play a key role in the formation of aortic aneurysm in patients with bicuspid and tricuspid aortic valves [32]. Additionally, Martin *et al*. [33] have demonstrated that decreased soluble guanylyl cyclase (sGC) activity in aortic aneurysms was associated with increased expression of abnormal sGC splicing variants, suggesting that AS contributes to diminished sGC function in vascular dysfunction. Similarly, APA is a ubiquitous mechanism in eukaryotic cells and is crucial for diverse cellular processes, such as mRNA metabolism, cell proliferation and differentiation, protein localization and diversification, and more commonly in gene regulation [34]. Importantly, some studies have revealed that it plays a fundamental role in the establishment of human diseases. For example, in the failing heart, the 3′-end formation of numerous mRNAs is changed, corresponding to the decrease of poly(A)-binding protein nuclear-1 expression [35]. APA contributes to cardiomyocyte hypertrophy via changing the expression of hypertrophy genes [36]. This evidence suggests that specific APA events may participate in the development of cardiovascular disease. Regrettably, there is almost no relevant research on APA in AAA. In our study, AS, APA and SSR events were initially identified in two groups, and future studies that address the pathophysiological consequences of these events are needed to evaluate their role in the pathogenesis of AAA and whether manipulation of these changes can be considered a therapeutic option for AAA.

LncRNAs are considered to be engaged in numerous vital biological processes as a crucial regulator of gene expression [37]. The third generation of nanopore RNA sequencing is helpful to identify the genetic structure of lncRNAs. Previous studies have shown that several essential lncRNAs may be involved in regulating the progression of AAA [38]. In the current study, we determined the comprehensive expression profile of lncRNAs in the two groups and proved ONT methods were suitable for the identification of lncRNAs.

In summary, third-generation nanopore-based RNA sequencing was introduced to explore the regulatory mechanisms of AAA. Especially, the study represented the initial comprehensive analysis of AS, APA and SSR events in AAA. These findings may provide novel insights into the pathogenesis of AAA, and future research should address the pathophysiological consequences of these changes in order to assess their role in the pathogenesis of AAA, and whether manipulation of these changes can be considered as a treatment option for AAA.

## Data accessibility

This article has no additional data.

## References

[1] Golledge J. 2019 Abdominal aortic aneurysm: update on pathogenesis and medical treatments. Nat. Rev. Cardiol. **16**, 225-242. (10.1038/s41569-018-0114-9) 30443031

[2] Sakalihasan N, Limet R, Defawe OD. 2005 Abdominal aortic aneurysm. Lancet **365**, 1577-1589. (10.1016/S0140-6736(05)66459-8) 15866312

[3] Li DY et al. 2018 H19 induces abdominal aortic aneurysm development and progression. Circulation **138**, 1551-1568. (10.1161/CIRCULATIONAHA.117.032184) 29669788 PMC6193867

[4] Lu H et al. 2020 Cyclodextrin prevents abdominal aortic aneurysm via activation of vascular smooth muscle cell transcription factor EB. Circulation **142**, 483-498. (10.1161/CIRCULATIONAHA.119.044803) 32354235 PMC7606768

[5] Investigators MRS. 2017 Aortic wall inflammation predicts abdominal aortic aneurysm expansion, rupture, and need for surgical repair. Circulation **136**, 787-797. (10.1161/CIRCULATIONAHA.117.028433) 28720724 PMC5571881

[6] Karthikesalingam A, Vidal-Diez A, Holt PJ, Loftus IM, Schermerhorn ML, Soden PA, Landon BE, Thompson MM. 2016 Thresholds for abdominal aortic aneurysm repair in England and the United States. N. Engl. J. Med. **375**, 2051-2059. (10.1056/NEJMoa1600931) 27959727 PMC5177793

[7] Visscher PM, Wray NR, Zhang Q, Sklar P, Mccarthy MI, Brown MA, Yang J. 2017 10 Years of GWAS discovery: biology, function, and translation. Am. J. Hum. Genet. **101**, 5-22. (10.1016/j.ajhg.2017.06.005) 28686856 PMC5501872

[8] Calkin AC, Tontonoz P. 2010 Genome-wide association studies identify new targets in cardiovascular disease. Sci. Transl. Med. **2**, 48ps46. (10.1126/scitranslmed.3001557) 20826839

[9] Kessler T, Vilne B, Schunkert H. 2016 The impact of genome-wide association studies on the pathophysiology and therapy of cardiovascular disease. EMBO Mol. Med. **8**, 688-701. (10.15252/emmm.201506174) 27189168 PMC4931285

[10] Wahlgren CM, Larsson E, Magnusson PKE, Hultgren R, Swedenborg J. 2010 Genetic and environmental contributions to abdominal aortic aneurysm development in a twin population. J. Vasc. Surg. **51**, 3-7; discussion 7. (10.1016/j.jvs.2009.08.036) 19939604

[11] Vandestienne M et al. 2021 TREM-1 orchestrates angiotensin II-induced monocyte trafficking and promotes experimental abdominal aortic aneurysm. J. Clin. Invest. **131**, e142468. (10.1172/JCI142468) PMC7810476 33258804

[12] Raffort J, Lareyre F, Hassen-Khodja R, Chinetti G, Mallat Z. 2017 Monocytes and macrophages in abdominal aortic aneurysm. Nat. Rev. Cardiol. **14**, 457-471. (10.1038/nrcardio.2017.52) 28406184

[13] Robertson L, Atallah E, Stansby G. 2017 Pharmacological treatment of vascular risk factors for reducing mortality and cardiovascular events in patients with abdominal aortic aneurysm. Cochrane Database Syst. Rev. **1**, CD010447. 28079254 10.1002/14651858.CD010447.pub3 PMC6464734

[14] Moreno DH, Cacione DG, Baptista-Silva JC. 2018 Controlled hypotension versus normotensive resuscitation strategy for people with ruptured abdominal aortic aneurysm. Cochrane Database Syst. Rev. **6**, CD011664. 29897100 10.1002/14651858.CD011664.pub3 PMC6513606

[15] Harrison SC et al. 2018 Genetic association of lipids and lipid drug targets with abdominal aortic aneurysm: a meta-analysis. JAMA Cardiol. **3**, 26-33. (10.1001/jamacardio.2017.4293) 29188294 PMC5833524

[16] Eckstein HH, Maegdefessel L. 2020 Linking obesity with abdominal aortic aneurysm development. Eur. Heart J. **41**, 2469-2471. (10.1093/eurheartj/ehz882) 31848586 PMC7340355

[17] Sakalihasan N, Michel JB, Katsargyris A, Kuivaniemi H, Defraigne JO, Nchimi A, Powell JT, Yoshimura K, Hultgren R. 2018 Abdominal aortic aneurysms. Nat. Rev. Dis. Primers **4**, 34. (10.1038/s41572-018-0030-7) 30337540

[18] Pinard A, Jones GT, Milewicz DM. 2019 Genetics of thoracic and abdominal aortic diseases. Circ. Res. **124**, 588-606. (10.1161/CIRCRESAHA.118.312436) 30763214 PMC6428422

[19] Nyberg A et al. 2008 Abdominal aortic aneurysm and cytomegalovirus infection. J. Med. Virol. **80**, 667-669. (10.1002/jmv.21022) 18297722

[20] Edvinsson M et al. 2010 Persistent Chlamydophila pneumoniae infection in thoracic aortic aneurysm and aortic dissection? Ups J. Med. Sci. **115**, 181-186. (10.3109/03009731003778719) 20384541 PMC2939519

[21] Matsui S, Hatta T. 2011 Mycotic abdominal aortic aneurysm in a dialysis patient with catheter-related methicillin-resistant *Staphylococcus aureus* bacteremia. Ther. Apher. Dial. **15**, 113-114. (10.1111/j.1744-9987.2010.00854.x) 21272261

[22] Uchiyama-Tanaka Y, Mori Y. 2006 Miliary tuberculosis with hypercalcemia, and a false abdominal aortic aneurysm, but no pulmonary findings. Intern. Med. **45**, 1297-1302. (10.2169/internalmedicine.45.1740) 17170504

[23] Forbes TL, Harris JR, Nie RG, Lawlor DK. 2004 Tuberculous aneurysm of the supraceliac aorta–a case report. Vasc. Endovasc. Surg. **38**, 93-97. (10.1177/153857440403800113) 14760484

[24] Kimura H. 2006 Pathogenesis of chronic active Epstein-Barr virus infection: is this an infectious disease, lymphoproliferative disorder, or immunodeficiency? Rev. Med. Virol. **16**, 251-261. (10.1002/rmv.505) 16791843

[25] Xiao H, Hu B, Luo R, Hu H, Zhang J, Kuang W, Zhang R, Li L, Liu G. 2020 Chronic active Epstein-Barr virus infection manifesting as coronary artery aneurysm and uveitis. Virol. J. **17**, 166. (10.1186/s12985-020-01409-8) 33121509 PMC7597064

[26] Chimenti C, Verardo R, Grande C, Francone M, Frustaci A. 2020 Infarct-like myocarditis with coronary vasculitis and aneurysm formation caused by Epstein-Barr virus infection. ESC Heart Fail **7**, 938-941. (10.1002/ehf2.12611) 32187886 PMC7261578

[27] Luo CY, Ko WC, Tsao CJ, Yang YJ, Su IJ. 1999 Epstein-Barr virus-containing T-cell lymphoma and atherosclerotic abdominal aortic aneurysm in a young adult. Hum. Pathol. **30**, 1114-1117. (10.1016/S0046-8177(99)90232-0) 10492049

[28] Yu X, Yu K, Chen B, Liao Z, Qin Z, Yao Q, Huang Y, Liang J, Huang W. 2021 Nanopore long-read RNAseq reveals regulatory mechanisms of thermally variable reef environments promoting heat tolerance of scleractinian coral *Pocillopora damicornis*. Environ. Res. **195**, 110782. (10.1016/j.envres.2021.110782) 33503412

[29] Wen WX, Mead AJ, Thongjuea S. 2020 Technological advances and computational approaches for alternative splicing analysis in single cells. Comput. Struct. Biotechnol. J. **18**, 332-343. (10.1016/j.csbj.2020.01.009) 32099593 PMC7033300

[30] Song Y, Botvinnik OB, Lovci MT, Kakaradov B, Liu P, Xu JL, Yeo GW. 2017 Single-cell alternative splicing analysis with expedition reveals splicing dynamics during neuron differentiation. Mol. Cell **67**, 148-161. (10.1016/j.molcel.2017.06.003) 28673540 PMC5540791

[31] Hoque M, Ji Z, Zheng D, Luo W, Li W, You B, Park JY, Yehia G, Tian B. 2013 Analysis of alternative cleavage and polyadenylation by 3′ region extraction and deep sequencing. Nat. Methods **10**, 133-139. (10.1038/nmeth.2288) 23241633 PMC3560312

[32] Kjellqvist S et al. 2013 A combined proteomic and transcriptomic approach shows diverging molecular mechanisms in thoracic aortic aneurysm development in patients with tricuspid- and bicuspid aortic valve. Mol. Cell Proteomics **12**, 407-425. (10.1074/mcp.M112.021873) 23184916 PMC3567863

[33] Martin E, Golunski E, Laing ST, Estrera AL, Sharina IG. 2014 Alternative splicing impairs soluble guanylyl cyclase function in aortic aneurysm. Am. J. Physiol. Heart Circ. Physiol. **307**, H1565-H1575. (10.1152/ajpheart.00222.2014) 25239802 PMC4255009

[34] Tian B, Manley JL. 2017 Alternative polyadenylation of mRNA precursors. Nat. Rev. Mol. Cell Biol. **18**, 18-30. (10.1038/nrm.2016.116) 27677860 PMC5483950

[35] Creemers EE et al. 2016 Genome-wide polyadenylation maps reveal dynamic mRNA 3'-end formation in the failing human heart. Circ. Res. **118**, 433-438. (10.1161/CIRCRESAHA.115.307082) 26671978

[36] Soetanto R et al. 2016 Role of miRNAs and alternative mRNA 3'-end cleavage and polyadenylation of their mRNA targets in cardiomyocyte hypertrophy. Biochim. Biophys. Acta **1859**, 744-756. (10.1016/j.bbagrm.2016.03.010) 27032571

[37] He X, Lian Z, Yang Y, Wang Z, Fu X, Liu Y, Li M, Tian J, Yu T, Xin H. 2020 Long non-coding RNA PEBP1P2 suppresses proliferative VSMCs phenotypic switching and proliferation in atherosclerosis. Mol. Ther. Nucleic Acids **22**, 84-98. (10.1016/j.omtn.2020.08.013) 32916601 PMC7490454

[38] Tian L, Hu X, He Y, Wu Z, Li D, Zhang H. 2018 Construction of lncRNA-miRNA-mRNA networks reveals functional lncRNAs in abdominal aortic aneurysm. Exp. Ther. Med. **16**, 3978-3986. (10.3892/etm.2018.6690) 30344676 PMC6176170

